# Assessment of the relationship between living alone and the risk of depression based on longitudinal studies: A systematic review and meta-analysis

**DOI:** 10.3389/fpsyt.2022.954857

**Published:** 2022-08-30

**Authors:** Daolin Wu, Fuwei Liu, Shan Huang

**Affiliations:** ^1^Department of Sleep Medicine, Ganzhou People’s Hospital, Ganzhou, China; ^2^Department of Cardiology, Ganzhou People’s Hospital, Ganzhou, China; ^3^Department of Psychiatry, The Third People’s Hospital of Ganzhou, Ganzhou, China

**Keywords:** living alone, depression, longitudinal studies, systematic review, meta-analysis

## Abstract

**Background:**

Living alone is one of the most common psychosocial factors that may have an impact on lifestyle management and health status. Although many previous cross-sectional studies have found that living alone increases the risk of depression. However, this risk has rarely been assessed on the basis of longitudinal studies. Therefore, we will explore this relationship on the basis of longitudinal studies.

**Methods:**

We systematically searched Pubmed, Embase, and Cochrane databases up to May 2022. Adjusted odds ratios (ORs), and 95% confidence intervals (CIs) were pooled by a random-effects model using an inverse variance method.

**Results:**

Seven studies (six cohort studies and one case-control study) were included in our study. A total of 123,859 without a history of psychosis individuals were included, and the proportion of females was 65.3%. We applied a random-effects model to minimize the heterogeneity. Overall, the pooled data suggest that people living alone are associated with an increased risk of depression compared to those who do not live alone (OR 1.42, 95%CI 1.19–1.70).

**Conclusion:**

Compared to people who live with others, living alone increases the risk of depression. Only cross-sectional studies and a few longitudinal studies currently support this association; more high-quality studies will be required in the future to confirm this causal association.

## Introduction

With declining birth rates and an overall increase in average life expectancy, the world’s population has been aging in recent years. Social living arrangements have changed significantly, particularly with the increasing number of single-person households (1), with around 30% of people in Western countries living alone and a significantly higher proportion of middle-aged and older people than previously (2, 3). Due to the global COVID-2019 epidemic, the impact of social isolation disadvantages has been rising in recent years (4). Social isolation is a substantial risk factor for physical health, cardiovascular disease, stroke, and premature mortality in studies (5–7). Living alone is also a prevalent psychosocial issue and an essential component of negative social variables. Living alone increases the chances of social isolation. Living alone may be harmful to one’s mental health, especially among older people. Previous research has found that persons who live alone have poorer mental health and a lower quality of life than those who live with others. Additionally, loneliness can contribute to depression (8). Unfavorable socioeconomic situations are determinants of social health, and poor social conditions (living alone, low income, little social interaction, and a lack of care) raise the risk of depression in the population, particularly among the elderly (9–11). Depression, one of the main causes of disability worldwide, is a mental health disorder characterized by chronic sorrow, loss of interest or pleasure in formerly valued or pleasant activities, sleep and eating difficulties, exhaustion, and poor concentration (12, 13). A meta-analysis reported that the prevalence of late-life depression in China was 22.7% (14). According to research, depression is linked to the incidence of cardiovascular illness, stroke, coronary heart disease, gastrointestinal problems, hypertension, asthma, arthritis, disability, suicide, and self-harm. Depression not only harms the health of older folks but also lowers the quality of life for individuals and their families (15–17). Many previous cross-sectional studies have found that living alone increases the risk of developing depression. A meta-analysis that included cross-sectional studies showed that living alone increased the risk of depression by 44% (18). However, previous studies have some limitations: (a) the majority of them are cross-sectional studies with methodological limitations; (b) meta-analyses that included cross-sectional studies did not include longitudinal studies, including cohort studies and RCT studies, which failed to truly reflect the causal relationship between solitary living and depression; and (c) the results are controversial; based on these limitations, we will include longitudinal studies to further explore this relationship.

## Methods

### Literature search

The PRISMA (Preferred Reporting Items for Systematic Reviews and Meta-Analyses) project was used to conduct this meta-analysis and systematic review (19). We did not need to obtain ethical approval because the results of the studies included in this meta-analysis were already published. We systematically searched the electronic databases PubMed, Embase, and Cochrane for relevant studies reporting the relationship between living alone and the risk of diabetes until May 2022. The search strategy used the Boolean operators ‘and’ in combination with two search terms: (1) social isolation OR living alone OR living status OR living arrangement (2) depression OR mental health problems. We did not apply language restrictions in the literature search. To ensure the comprehensiveness of the literature search, we screened the reference lists of the retrieved studies to identify additional reports.

### Eligibility criteria

The following studies were included: (1) The study included participants who did not have depression. (2) Comparison: living alone versus living with others (3) Outcome: risk of depression (4) Study design: randomized controlled trials or cohort studies (5) Estimates of effect: adjusted odds ratios (ORs) and 95 percent confidence intervals (CIs). We excluded publications that did not include effect estimates, such as reviews, case reports, case series, editorials, letters, cross-sectional studies, guidelines, and conference abstracts. If subjects from different studies came from the same data source, the study with the longest duration or largest sample size was included.

### Study selection and data extraction

Two independent authors reviewed all of the retrieved literature (DL-W and FW-L). Based on pre-defined criteria, we read the titles and abstracts of potentially eligible studies before reviewing them in depth. Negotiation was used to settle disagreements between the two authors. We collected information on the following topics for each study: first author; year of publication; study design; study site; data source; follow-up time; primary baseline information; and adjusted ORs, adjusted for confounders. If a study reported adjusted ORs in multiple models, only the most adjusted model was used.

### Study quality assessment

To measure study quality, we employed a modified Newcastle-Ottawa Scale (NOS). This scale has three components: cohort selection, cohort comparability, and outcome evaluation. studies with NOS scores < 6 were defined as low quality, and studies with NOS scores ≥ 6 were defined as moderate to high quality (20).

### Data analysis

Review Manager 5.3 software was used for all statistical analyses (the Nordic Cochrane Center, Rigshospitalet, Denmark). The Cochrane Q test and the I^2^ statistic were the two most often used statistical techniques to quantify heterogeneity, with *P* < 0.1 and *I*^2^ > 50% indicating significant heterogeneity, respectively. The natural logarithm of the OR and standard errors of the included studies were determined and then pooled using an inverse variance random effects model.

## Results

**FIGURE 1:**
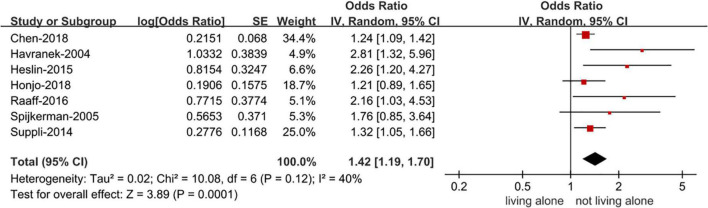
Forest plot for the association of living alone with the risk of depression. CI, confidence interval; SE, standard error; IV, inverse of the variance.

Supplementary Table 1 depicts the procedure of doing a literature search. Electronic searches in the PubMed, Embase and Cochrane databases yielded 2056 studies. Through titles and abstracts, full text reading, and reference to predetermined criteria, we finally included seven studies (Supplementary Figure 1). All of the included studies had NOS values ranging from moderate to high (Supplementary Table 2). The baseline characteristics of the included studies are shown in Table 1. The meta-analysis includes six cohort studies (21–26) and one case-control study (27). We gathered baseline characteristics from the included studies, such as authors, study type, data source, geographic region, sample size, study population, mean age, male proportion, length of follow-up, and adjusted confounders. There were 123,859 people without a history of psychosis in the study, with 65.3 percent of them being female. To reduce heterogeneity, we used a random-effects model. Overall, the pooled data showed that living alone was associated with a higher risk of depression than not living alone (OR 1.42, 95% CI 1.19–1.70) (Figure 1).

**TABLE 1:** Baseline patient characteristics of the included studies.

Included studies	Study design	Data source	Region	Sample size	Population	Age (mean, y)	Female (N, %)	Living alone (N, %)	Depression (N, %)	Follow-up time (year)	Adjusted confounding factors	Nos.
Heslin et al. (27)	Case–control study	AESOP study	London	896	Without a history of psychosis	NA	383 (42.7)	NA	51 (3.9)	8	Gender, age, center and ethnicity and 95% CIs for follow-up diagnosis of PMD, schizophrenia and bipolar compared with controls	7
Chen et al. (21)	Retrospective cohort study	The Taiwan National Health Insurance Research Database	Taiwan	43,885	Glaucoma patients and age and gender-matched control subjects without glaucoma	59.4	22,005 (50.1)	4,082 (9.3)	518 (10.3)	11	Glaucoma, age, gender, Charlson comorbidity index, insurance cost, urbanization level of residency, living alone, and substance abuse	8
Havranek et al. (23)	Prospective cohort study; multicenter	The KCCQ interpretability Study	The U.S. and Canada	245	Outpatients with heart failure and without depressive symptoms	62.5	62 (25.4)	65 (26.6)	52 (21.2)	1	Medical Outcomes Study-Depression Questionnaire	8
Spijkerman et al. (24)	Prospective cohort study	The depression after myocardial infarction study	The north of the Netherlands	882	Patients with post-myocardial infarction	60 (male) vs. 63.70 (female);	148 (16.8)	123 (14)	NA	1	Gender, age, living alone, education, maximum CK, maximum CK MB, LVEF, anterior site, Killip class, and history of MI; cardiologic treatment post-MI, complications during hospitalization, exercise tolerance, and duration of hospital stay in days; history of depressive disorder and vital exhaustion; pre-MI	6
de Raaff et al. (22)	Prospective cohort study	A hospital databases	Netherlands	139	Curative mastectomy for breast cancer without relapse	65.6	139 (100)	71 (51.1)	67 (48.2)	6	The variables duration of follow-up, type of household, educational level, comorbidity, polypharmacy and adjuvant hormonal/endocrine therapy	7
Suppli et al. (25)	Prospective cohort study	Death and emigration in the Central Population Registry	Denmark	35,643	Breast Cancer	NA	35,643 (100)	NA	NA	13	Age, Menopausal Status, and Calendar Period	8
Honjo et al. (26)	Prospective cohort study	JAGES	Japan	42,169	Older adults	72.6	22,513 (53.4)	4,338 (10.3)	5,474 (13.0)	2.6	Age, GDS score at baseline, age group, years of educational attainment), equivalent household income groups, employment status, receiving treatment for any disease, poor self-rated health, time spent walking per day, and residential area	8

AESOP, etiology and ethnicity in schizophrenia and other psychoses; PMD, psychotic major depression; KCCQ, the Kansas City Cardiomyopathy Questionnaire; MI, myocardial infarction; JAGES, the Japan Gerontological Evaluation Study.

We performed sensitivity analyses by excluding one study at a time (Supplementary Table 3). ORs ranged between 1.33 and 1.58. Heterogeneity results ranged between 31 and 50%. The main results were similar to those of the main analysis using the random-effects model. Furthermore, when we re-performed the analysis using the fixed-effects model (Supplementary Figure 2), the results did not change substantially.

### Publication bias

We did not examine publication bias since our meta-analysis comprised just six trials. It was emphasized that publication bias should not be addressed for some reported outcomes when less than ten studies were included.

## Discussion

Although previous meta-analyses have found that living alone increases the risk of depression, as they were only based on cross-sectional studies, with this limitation in mind, we will for the first time examine the relationship between living alone and the risk of depression from data from longitudinal studies. The main finding of our study is that living alone increases the risk of depression by 42%. This finding is consistent with the results of a previous meta-analysis (18).

At the time of statistical analysis, we found a moderate degree of heterogeneity in the findings, which may be related to the characteristics of the study populations included and residual confounding factors. a case-control study by Heslin et al. included 896 participants in London with no history of psychosis and showed that the risk of depression was 2.26 times higher in those who lived alone than in those who did not live alone (27). A Japanese cohort study that included 42,169 older adults found that living arrangements were associated with the risk of depressive symptoms in both men and women and that these associations varied by gender and level of community social cohesion. The study cautions that more attention needs to be paid to whether individuals live alone and with whom individuals live to prevent depressive symptoms in older adults (26). Patients who appear to have a disease state or history of the disease are at higher risk of developing depression compared to the general population, and the disease itself may affect the patient’s quality of life psychosocially. Two cohort studies of patients with breast cancer were included in our study, with the cohort study from Denmark finding a 70% increased risk of depression in the first year after diagnosis for living alone and a 3.09 times higher rate of antidepressant use than for non-living alone patients. The study suggested that women with co-morbidities, lymph node-positive disease, and those aged 70 years or older were at the highest risk. However, no clear association was found between the type of surgery or adjuvant treatment and the risk of depression (25). A single-center cohort study from the Netherlands showed that living alone increased the risk of depression in breast cancer patients by 2.16 times that of non-solitary patients, and previous studies have found that breast cancer reconstructive therapy reduces the risk of depression, but no such association was found in this study (22). A community-based retrospective cohort research in Taiwan discovered that glaucoma patients living alone had a considerably higher incidence of depression than the general population. Age, gender, poor income, substance usage, and living alone were all major risk factors for developing depression in glaucoma patients (21). Depressive symptoms after myocardial infarction appear to be driven primarily by the psychological and social consequences of myocardial infarction in patients prone to depression, particularly a history of depression or the risk of developing depression in patients exacerbated by the presence of vital failure (24). Another cohort study incorporating heart failure suggests that social factors and health status predict the development of depression in heart failure outpatients, prompting clinicians to identify patients at high risk for early psychosocial intervention (23).

Living alone raises the risk of developing depression, but it doesn’t stop there. Numerous previous cross-sectional studies have also discovered that this risk varies by age, gender, and location. Older people living alone are more vulnerable than younger people (28), men living alone are more vulnerable than women (29), and rural people living alone are more vulnerable than urban people (30). Unfortunately, the number of longitudinal studies currently available is insufficient to allow for subgroup analysis in our study.

As the worldwide birth rate falls and the global aging rate rises, more and more individuals are adjusting their lifestyles to accommodate. The amount of persons living alone has increased dramatically in Western countries, reaching 30%, with the older population being particularly vulnerable (2). Because of changing family arrangements and urbanization, the number of seniors living alone is continuously increasing in developed Asian nations such as Korea, Japan, and China (31). The negative impacts of living alone are becoming a major issue. Living alone leads to a lack of contact with others and atrophy of social networks, exacerbating social disconnection and leading to objective social isolation. There is growing concerned about the adverse effects of living alone, and previous research evidence suggests that social isolation is associated with an increased risk of physical (e.g., cardiovascular disease and diabetes) and mental health (e.g., anxiety and depression) disorders (32, 33). Depression is now one of the leading chronic diseases and is also considered a major public health problem, and may become the second most common cause of disability by 2020, after heart disease (34). Depression is associated with genetics (35), behavior, psychological factors (36), sleep quality (37), and health status. Social support is an important factor in depression. Living arrangements as a structural element of social support may help reduce and prevent the risk of depression in older adults. There are several possible explanations for the increased risk of depression associated with living alone: first, people who live alone have poorer economic and material conditions and less social support; second, people who live alone lack contact with others and may have poorer health habits and lower levels of mental health awareness; and third, people who live alone are at higher risk of developing diseases. More research are needed based on these factors to investigate the impact of psychosocial factors on people living alone and their prognosis, to identify at-risk patients early, and to intervene in the development of depression.

### Limitations

There are some limitations to the current study that should be highlighted. The number of includable longitudinal studies was small, and there was heterogeneity and residual confounding between studies; second, due to limited data, subgroup analyses for gender and urban-rural differences were not conducted; and third, there is a lack of relevant interventions to demonstrate a reduced risk of depression in people living alone. More long-term studies are needed in the future to validate this causal association, based on these limitations.

## Conclusion

Compared to people who live with others, living alone increases the risk of depression. Only cross-sectional studies and a few longitudinal studies currently support this association; more high-quality studies will be required in the future to confirm this causal association.

## Data availability statement

The original contributions presented in this study are included in the article/Supplementary material, further inquiries can be directed to the corresponding author/s.

## Author contributions

All authors listed have made a substantial, direct, and intellectual contribution to the work, and approved it for publication.
