# Supplementary material for: Assessment of the relationship between living alone and the risk of depression based on longitudinal studies: A systematic review and meta-analysis
Source: Front Psychiatry. 2022 Aug 30;13:954857. doi: 10.3389/fpsyt.2022.954857 (PMC9468273; doi:10.3389/fpsyt.2022.954857)
Supplement: Supplementary file 1 [file Data_Sheet_1.docx]

**Supplementary Table 1. The search strategies of this meta-analysis**

|  | **Search terms** | **No.** |
| --- | --- | --- |
| **PubMed** | | |
| #1 | depression OR mental health problems | 155144 |
| #2 | social isolation OR living alone OR living status OR living arrangement | 28673 |
| #3 | #1 and #2 | 1156 |
| **Embase** | | |
| #1 | depression | 573812 |
| #2 | mental health problems | 18975 |
| #3 | #1 OR #2 | 587295 |
| #4 | living alone | 6237 |
| #5 | social isolation | 13846 |
| #6 | living arrangement | 1032 |
| #7 | #4 OR #5 OR #6 | 1325493 |
| #8 | #3 and #7 | 1324 |
| **cochrane** | | |
| #1 | depression | 13893 |
| #2 | mental health problems | 7612 |
| #3 | #1 OR #2 | 20940 |
| #4 | social isolation OR living alone OR living status OR living arrangement | 13020 |
| #5 | #3 and #4 | 756 |

**Supplementary Figure 1. The process of the literature retrieval of this meta-analysis**


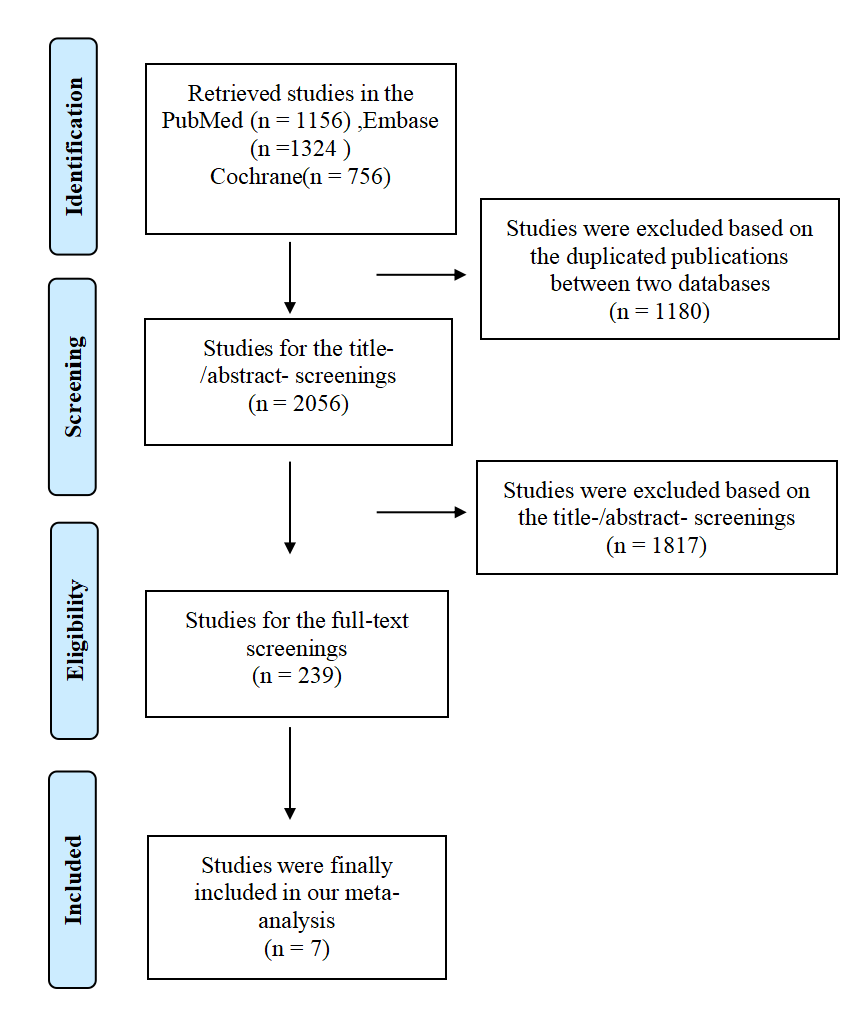


**Supplementary Figure 2. Forest plot for the association of living alone with the risk of depression using the fixed-effects model**


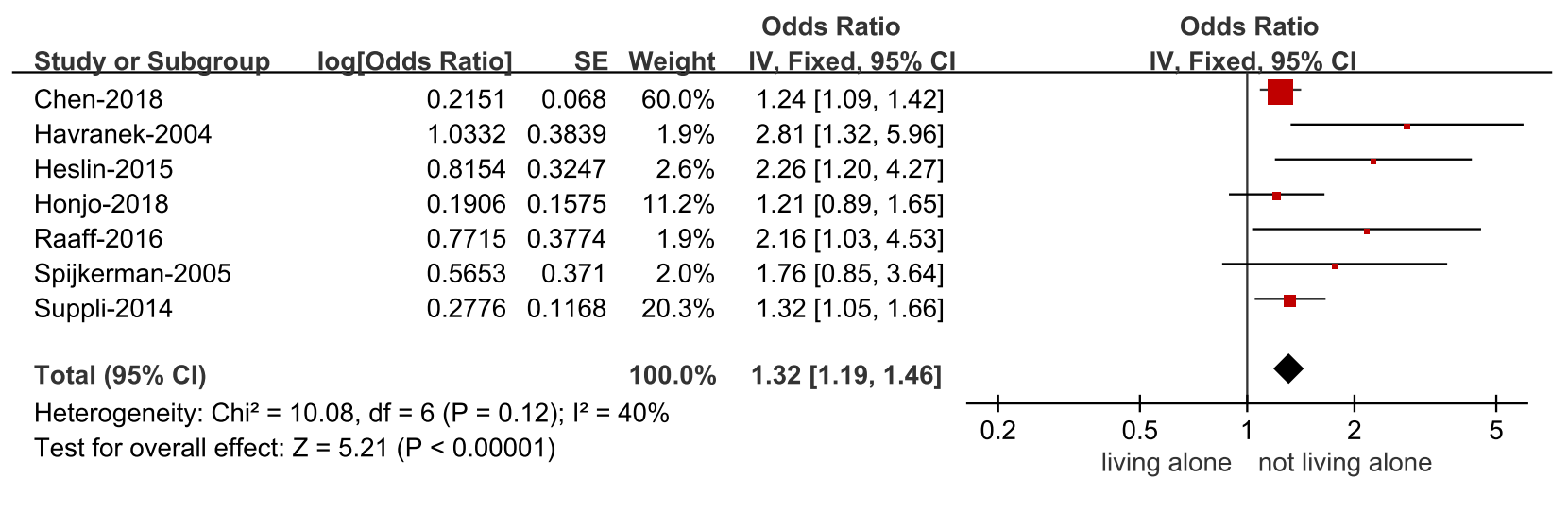


**Supplementary Table 2. Quality assessment for the included post-hoc analyses and observational studies**

| Included studies | Selection (0-4 points) | | | | Comparability (0-2 points) | | Outcome (0-3 points) | | | Total poitns* |
| --- | --- | --- | --- | --- | --- | --- | --- | --- | --- | --- |
|  | Representativeness of Exposed Cohort | Selection of Non-Exposed Cohort | Ascertainment of Exposure | Demonstration That Outcome of Interest Was Not Present at Start of Study | Adjust for the important Risk factors | Adjust for other risk factors | Assessment of outcome | Follow-up length | Loss to follow-up rate |  |
| Suppli-2014 | * | * | * | * | * | * | * | * |  | 8 |
| Spijkerman-2005 | * | * | * |  | * |  | * | * |  | 6 |
| Havranek-2004 | * | * | * | * | * | * | * | * |  | 8 |
| Chen-2018 | * | * | * | * | * | * | * | * |  | 8 |
| Raaff-2016 | * | * | * | * | * |  | * | * |  | 7 |
| Heslin-2015 | * | * | * | * | * |  | * | * |  | 7 |
| Honjo-2018 | * | * | * | * | * | * | * | * |  | 8 |

＊The Newcastle-Ottawa Scale (NOS) items, with a total score of 9 points, were used to evaluate the quality of the post-hoc analyses of RCTs and observational study which involve the selection of cohorts (0-4 points), the comparability of cohorts (0-2 points), and the assessment of the outcome (0-3 points)

**Supplementary Table 3. The heterogeneity of the included studies through sensitivity analysis**

| **Excluded study arm** | **HR (95% CI)** | **I^2^ (%)** | **p value** |
| --- | --- | --- | --- |
| Suppli-2014 | 1.55 (1.20 - 2.00) | 50 | 0.0009 |
| Spijkerman-2005 | 1.42 (1.17 - 1.71) | 47 | 0.0003 |
| Havranek-2004 | 1.33 (1.16 - 1.52) | 18 | <0.0001 |
| Chen-2018 | 1.58 (1.23 - 2.02) | 39 | 0.0003 |
| Raaff-2016 | 1.38 (1.16 - 1.65) | 40 | 0.0003 |
| Heslin-2015 | 1.35 (1.15 - 1.59) | 31 | 0.0002 |
| Honjo-2018 | 1.53 (1.22 - 1.91) | 49 | 0.0002 |
